# Proceedings of Patient Reported Outcome Measure’s (PROMs) Conference Oxford 2017: Advances in Patient Reported Outcomes Research: Oxford, UK. 8th June 2017

**DOI:** 10.1186/s12955-017-0757-y

**Published:** 2017-10-05

**Authors:** Galina Velikova, Jose M. Valderas, Caroline Potter, Laurie Batchelder, Christine A’Court, Matthew Baker, Jennifer Bostock, Angela Coulter, Ray Fitzpatrick, Julien Forder, Diane Fox, Louise Geneen, Elizabeth Gibbons, Crispin Jenkinson, Karen Jones, Laura Kelly, Michele Peters, Brendan Mulhern, Alexander Labeit, Donna Rowen, Keith Meadows, Jackie Elliott, John Brazier, Emma Knowles, Anju Keetharuth, John Brazier, Janice Connell, Jill Carlton, Lizzie Taylor Buck, Thomas Ricketts, Michael Barkham, Pushpendra Goswami, Sam Salek, Tatyana Ionova, Esther Oliva, Adele K. Fielding, Marina Karakantza, Saad Al-Ismail, Graham P. Collins, Stewart McConnell, Catherine Langton, Daniel M. Jennings, Roger Else, Jonathan Kell, Helen Ward, Sophie Day, Elizabeth Lumley, Patrick Phillips, Rosie Duncan, Helen Buckley-Woods, Ahmed Aber, Gerogina Jones, Jonathan Michaels, Ian Porter, Jaheeda Gangannagaripalli, Antoinette Davey, Ignacio Ricci-Cabello, Kirstie Haywood, Stine Thestrup Hansen, Jose Valderas, Deb Roberts, Anil Gumber, Bélène Podmore, Andrew Hutchings, Jan van der Meulen, Ajay Aggarwal, Sujith Konan, Andrew Price, William Jackson, Nick Bottomley, Michael Philiips, Toby Knightley-Day, David Beard, Elizabeth Gibbons, Ray Fitzpatrick, Joanne Greenhalgh, Kate Gooding, Elizabeth Gibbons, Chema Valderas, Judy Wright, Sonia Dalkin, David Meads, Nick Black, Carol Fawkes, Robert Froud, Dawn Carnes, Andrew Price, Jonathan Cook, Helen Dakin, James Smith, Sujin Kang, David Beard, The ACHE Study Team, Catrin Griffiths, Ella Guest, Diana Harcourt, Mairead Murphy, Sandra Hollinghurst, Chris Salisbury, Jill Carlton, Jackie Elliott, Donna Rowen, Anqi Gao, Andrew Price, David Beard, Agnieszka Lemanska, Tao Chen, David P. Dearnaley, Rajesh Jena, Matthew Sydes, Sara Faithfull, A. E. Ades, Daphne Kounali, Guobing Lu, Ines Rombach, Alastair Gray, Crispin Jenkinson, Oliver Rivero-Arias, Patricia Holch, Marie Holmes, Zoe Rodgers, Sarah Dickinson, Beverly Clayton, Susan Davidson, Jacqui Routledge, Julia Glennon, Ann M. Henry, Kevin Franks, Galina Velikova, Roma Maguire, Lisa McCann, Teresa Young, Jo Armes, Jenny Harris, Christine Miaskowski, Grigorios Kotronoulas, Morven Miller, Emma Ream, Elizabeth Patiraki, Alexander Geiger, Geir V. Berg, Adrian Flowerday, Peter Donnan, Paul McCrone, Kathi Apostolidis, Patricia Fox, Eileen Furlong, Nora Kearney, Chris Gibbons, Felix Fischer, Chris Gibbons, Joel Coste, Jose Valderas Martinez, Matthias Rose, Alain Leplege, Sarah Shingler, Natalie Aldhouse, Tamara Al-Zubeidi, Andrew Trigg, Helen Kitchen, Antoinette Davey, Ian Porter, Colin Green, Jose M. Valderas, Joanna Coast, Sarah Smith, Jolijn Hendriks, Nick Black, Koonal Shah, Oliver Rivero-Arias, Juan-Manuel Ramos-Goni, Simone Kreimeier, Mike Herdman, Nancy Devlin, Aureliano Paolo Finch, John E. Brazier, Clara Mukuria, Bernarda Zamora, David Parkin, Yan Feng, Andrew Bateman, Mike Herdman, Nancy Devlin, Thomas Patton, Nils Gutacker, Koonal Shah

**Affiliations:** 1 Leeds Institute of Cancer & Pathology, University of Leeds and Leeds Teaching Hospitals, Leeds, UK; 2 Health Services & Policy Research Group, University of Exeter Medical School, Exeter, UK; 3 Exeter Collaboration for Academic Primary Care (APEx), Exeter, UK; 4 University of Oxford, Oxford, UK; 5 University of Kent, Canterbury, UK; 6 QORU: Quality and Outcomes of Person-centered Care Policy Research Unit, Department of Health England, Canterbury, Kent UK; 7 NIHR Collaboration for Leadership in Applied Health Research and Care (CLAHRC) Oxford, Oxford, UK; 8 University of Sheffield, Sheffield, UK; 9 Oxford University Innovation, Oxford, UK; 10 University of Technology, Sydney, Sydney, Australia; 11 University of Sheffield, Sheffield, UK; 12 School of Life and Medical Sciences, University of Hertfordshire, Hatfield, Hertfordshire UK; 13 National Medical Surgical Centre and Multinational Centre for Quality of Life Research, St Petersburg, Russia; 14 Haematology Unit, Azienda Ospedaliera, Reggio Calabria, Italy; 15 UCL Cancer Institute, London, UK; 16 Leeds Teaching Hospitals NHS Trust, Leeds, UK; 17 Singleton Hospital, ABM University Health Board, Swansea, UK; 18 Oxford University Hospitals NHS Trust, Oxford, UK; 19 Royal Surrey County Hospital NHS Foundation Trust, Guildford, Surrey UK; 20 Patient Research Partner, Milton Keynes, UK; 21 Cardiff and Vale University Health Board, Cardiff, UK; 22 Imperial College London, London, UK; 23 Goldsmiths College, London, UK; 24 The University of Sheffield, Sheffield, UK; 25 Sheffield Teaching Hospitals NHS Foundation Trust, Sheffield, UK; 26 Leeds Beckett University, Leeds, UK; 27 University of Exeter Medical School, Devon, UK; 28 Nuffield Department of Primary Care Health Sciences, University of Oxford, Oxfordshire, UK; 29 Royal College of Nursing Research Institute, University of Warwick, Warwickshire, UK; 30 Department of Haematology, Zealand University Hospital, Køge, Denmark; 31 Department of Regional Health Research, University of Southern Denmark, Odense, Denmark; 32 Royal Liverpool & Broadgreen University Hospitals Trust, Merseyside, UK; 33 Centre for Health and Social Care Research, Sheffield Hallam University, Sheffield, UK; 34 London School of Hygiene & Tropical Medicine, London, UK; 35 The Royal College of Surgeons of England, London, UK; 36 University College London Hospitals NHS Foundation Trust, London, UK; 37 University of Oxford, Oxford, UK; 38 Nuffield Department of Population Health, University of Oxford, Oxford, UK; 39 University of Leeds, Leeds, UK; 40 University of Liverpool, Liverpool, UK; 41 University of Oxford, Oxford, UK; 42 University of Exeter, Exeter, UK; 43 London School of Hygiene and Tropical Medicine, London, UK; 44 Northumbria University, Newcastle-upon-Tyne, UK; 45 Barts and the London School of Medicine and Dentistry, London, UK; 46 Warwick Medical School Clinical Trials Unit, Coventry, UK; 47 Norges Helsehøyskole, Campus Kristiania, Oslo, Norway; 48 Univeristy of Oxford, Oxford, UK; 49 University of the West of England, Bristol, UK; 50 University of Bristol, Bristol, UK; 51 University of Sheffield, Sheffield, UK; 52 University of Oxford, Oxford, UK; 53 Univerity of Surrey, Guildford, UK; 54 Institute of Cancer Research and Royal Marsden NHS Trust, London, UK; 55 Cambridge University Hospitals, Addenbrookes Hospital, Cambridge, UK; 56 MRC Clinical Trials Unit at UCL, Institute of Clinical Trials and Methodology, London, UK; 57 University of Bristol, Avon, UK; 58 Nuffield Department of Population Health, University of Oxford, Oxford, UK; 59 Leeds Beckett Universty, Psychology Group, Leeds, West Yorkshire UK; 60 Univeristy of Leeds, Patient reported Outcomes Group, Leeds, West Yorkshire UK; 61 The Christie NHS Foundation Trust, Manchester, Lancashire UK; 62 Leeds Teaching Hospitals NHS Trust, Leeds, West Yorkshire UK; 63 University of Strathclyde, Glasgow, UK; 64 East & North Herts NHS Trust, Mount Vernon Cancer Centre, Northwood, UK; 65 Kings College, London, UK; 66 University of California, San Francisco, CA USA; 67 National & Kapodistrian University of Athens, Athens, Greece; 68 Medical University Vienna Comprehensive Cancer Centre, Vienna, Austria; 69 NTNU, Innlandet Hospital Trust, Lillehammer, Norway; 70 Docobo, Bookham, UK; 71 University of Dundee, Dundee, UK; 72 European Cancer Patient Coalition (ECPC), Brussels, Belgium; 73 University College Dublin School of Nursing, Dublin, Ireland; 74 University of Cambridge, Cambridge, UK; 75 Charité Universitätsmedizin Berlin, Berlin, Germany; 76 The Psychometrics Centre, University of Cambridge, Cambridge, UK; 77 APEMAC, EA 4360, Paris Descartes University and Epidemiology Unit, Hôtel Dieu, Assistance Publique, Hôpitaux de Paris, Paris, France; 78 University of Exeter Medical School, Exeter, UK; 79 Département d’Histoire et de Philosophie des Sciences Université Paris Diderot, Paris, France; 80 DRG Abacus, Oxfordshire, UK; 81 University of Exeter, Exeter, UK; 82 University of Bristol, Bristol, UK; 83 NIHR CLAHRC West, Bristol, UK; 84 London School of Hygiene & Tropical Medicine, London, UK; 85 Office of Health Economics, London, UK; 86 University of Sheffield, Sheffield, UK; 87 University of Oxford, Oxford, UK; 88 EuroQol Office, Rotterdam, The Netherlands; 89 University of Bielefeld, Bielefeld, Germany; 90 University of Sheffield, Sheffield, UK; 91 Office of Health Economics, London, UK; 92 Cambridgeshire Community Services NHS Trust, Cambridge, UK; 93 University of Cambridge, Cambridge, UK; 94 University of York, York, UK; 95 Office of Health Economics, London, UK; 96 University of Sheffield, Sheffield, UK

## Abstracts for plenary sessions

### P1 Using Patient Reported Outcome Measures (PROMs) in cancer care

#### Galina Velikova

##### Leeds Institute of Cancer & Pathology, University of Leeds and Leeds Teaching Hospitals, Leeds, UK

Monitoring of patients’ physical and psychological problems during and after cancer treatment is essential in modern oncology practice. Traditional clinical methods can be supplemented by Patient-Reported Outcomes Measures (PROMs) measures. The potential role of PROMs is recognised and endorsed by national and international practice guidelines. The introduction of formal measurement of PROMs in clinical practice is a complex health care innovation requiring careful planning, design and successful implementation of a number of essential components, such as choosing the patient questionnaire(s), a convenient affordable electronic method for reporting and display in hospital records and engaging clinicians to use and act on the reports. There is mounting research evidence that using PROMs in individual patient care in oncology is beneficial to patients, but this approach has not found a place in routine clinical practice. A brief overview of this evidence will be provided. Following this, the presentation will focus on examples of incorporating PROMs and eHealth interventions into routine patient care during and after cancer treatment, drawing on 20 years’ experience in Leeds of using electronic systems for capturing patient reported data in oncology settings. Examples will be given of: 1) Monitoring toxicity during systemic cancer treatment using online PROMs integrated with Electronic Patient Records (randomized trial part of NIHR eRAPID programme); 2) Service development project - Remote follow-up of testicular cancer patients using online PROMs plus community-based investigations. Examples of other online PROMs systems will be presented. The values and challenges of PROMs integration in routine oncology practice will be discussed.

### P2 The National Institutes of Health Patient-Reported Outcomes Measurement Information System (PROMIS): a view from the UK

#### Jose M Valderas^1,2^

##### ^1^Health Services & Policy Research Group, University of Exeter Medical School, Exeter UK; ^2^Exeter Collaboration for Academic Primary Care (APEx), Exeter, UK

The Patient-Reported Outcomes Measurement Information System (PROMIS) is a system for the measurement of patient reported outcomes whose development was funded by the US National Institute for Health. Its methodological rigor, scope, ambition and flexibility (including both standardized short forms and computerized adaptive administration) has turned it into one of the standards of PROMs measurement, although its use is still very limited outside the US. The presentation will provide an overview of the rationale for the development of the system, the methods employed in its development and the resulting scales and short forms and key characteristics, including Assessment Centre, the online platform supporting the use of PROMIs scales. Current applications and use of PROMIS in the UK will be reviewed and the potential for its application to support the management of patients in the NHS will be considered.

## Abstracts for oral papers

### Session 1a. Development and validation of measures - 1

#### A66 Validation of the Long-Term Conditions Questionnaire (LTCQ) in a diverse sample of health and social care users in England

##### Caroline Potter^1,4^, Laurie Batchelder^2,3^, Christine A’Court^1^, Matthew Baker^3^, Jennifer Bostock^3^, Angela Coulter^1,3^, Ray Fitzpatrick^1,3,4^, Julien Forder^2,3^, Diane Fox^2,3^, Louise Geneen^1,3^, Elizabeth Gibbons^1,4^, Crispin Jenkinson^1,3^, Karen Jones^2,3^, Laura Kelly^1,4^ & Michele Peters^1,3^

###### ^1^University of Oxford, Oxford, UK; ^2^University of Kent, Canterbury, UK; ^3^QORU: Quality and Outcomes of Person-centered Care Policy Research Unit, Department of Health England, Canterbury, Kent, UK; ^4^NIHR Collaboration for Leadership in Applied Health Research and Care (CLAHRC) Oxford, Oxford, UK

####### **Correspondence:** Caroline Potter

**Background:** Long-term conditions (LTCs) have emerged as a significant challenge to the sustainability of health systems. The rise of multi-morbidity has highlighted the need for joined-up services that can enhance people’s capacity for living well with LTCs.

**Aims:** The objective of this study was to validate a new generic well-being measure, the Long-Term Conditions Questionnaire (LTCQ).

**Methods:** Data were collected through two postal surveys (main and follow-up) from February 2016 to January 2017. The study sample included 1) a health care cohort of patients with at least one of 11 specified LTCs recruited through primary care practices and 2) a social care cohort receiving support for at least one LTC recruited through Local Authorities. The LTCQ’s construct validity was tested with reference to the EQ-5D (5-level version), Self-efficacy for Managing Chronic Disease scale, an Activities of Daily Living scale, and the Bayliss multi-morbidity scale. The follow-up survey enabled assessment of test-retest reliability.

**Results:** The total sample (N = 1,211; 23% response rate; 24% confirmed social care users) exhibited high multi-morbidity, with 93% reporting two or more LTCs and 43% reporting a mental health condition. Low levels of missing data indicate acceptability of the LTCQ within this diverse sample. The LTCQ exhibits high internal consistency (Cronbach’s α = 0.95) across the scale’s 20 items and excellent test-retest reliability (ICC = 0.94, 95% CI 0.93 to 0.95). Associations between the LTCQ and all reference measures were moderate to strong and in the expected directions, indicating convergent construct validity.

**Conclusions:** This study provides evidence for the reliability and validity of the Long-Term Conditions Questionnaire, which has potential for use in a variety of health and social care settings. The LTCQ could meet a need for an outcome measure that goes beyond symptoms and physical function, providing a more holistic metric for living well with LTCs.

#### A15 Developing preference based measures for diabetes for calculating QALYs: DHP-3D and DHP-5D

##### Brendan Mulhern^3^, Alexander Labeit^1^, Donna Rowen^1^, Keith Meadows^2^, Jackie Elliott^1^, John Brazier^1^ & Emma Knowles^1^

###### ^1^University of Sheffield, Sheffield, UK; ^2^Oxford University Innovation, Oxford, UK; ^3^University of Technology, Sydney, Sydney, Australia

####### **Correspondence:** John Brazier

**Background:** The cost per quality adjusted life year (QALY) of new interventions in long term conditions (LTCs) is assessed to inform resource allocation decisions. However, generic preference-based measures of health like EQ-5D may not be sufficiently sensitive for some LTCs. Condition specific PROMS like the Diabetes Health Profile (DHP) offer more relevant descriptions of how LTCs impact on people’s lives.

**Aims:** The aim was to develop two diabetes specific preference based measures (DHP-3D/DHP-5D) for use in the calculation of QALYs based on the non-preference based DHP (DHP-18/DHP-1).

**Methods:** For DHP-3D, Exploratory Factor Analysis and Rasch analyses were used to understand the dimensionality of the DHP-18, and select items to represent each in the classification system. The DHP-5D built on the DHP-3D adding two dimensions to extend the range of diabetes related impacts assessed. Each was valued by 150 members of the UK general public using the Time Trade Off (TTO) preference elicitation method. The valuation data was modelled using individual and mean level multivariate regression to produce utility values for every health state described.

**Results:** The DHP-3D classification system included three dimensions in line with the structure of the DHP-18 defined as mood, eating and social limitations, and the DHP-5D also included dimensions for hypoglycemic attacks and vitality. For both, the random effects generalized least squares regression model produced the most valid utility value set, with the DHP-3D and DHP-5D ranging from 0.983 (best state) to 0.717(worst state), and 0.979 to 0.618 respectively. The addition of the two extra dimensions leads to significant differences for the more severe levels of each matched dimension.

**Conclusions:** We have developed two diabetes specific preference based measures that, subject to psychometric assessment, can be used to provide condition specific utility values to complement generic utilities from more widely validated measures such as the EQ-5D.

#### A62 Constructing and Validating the Short Recovering Quality of Life (ReQoL) Measure for Use in a Mental Health Population

##### Anju Keetharuth, John Brazier, Janice Connell, Jill Carlton, Lizzie Taylor Buck, Thomas Ricketts & Michael Barkham

###### University of Sheffield, Sheffield, UK

####### **Correspondence:** Anju Keetharuth

**Background:** The concept of recovery for people experiencing mental health difficulties has received greater emphasis recently, prompting demands for new measures.

**Aims:** To assess the outcomes of mental health services in terms of recovering quality of life, we constructed and validated a 10-item version ReQoL measure.

**Methods:** After having established seven themes, we generated a long list of potential items which were tested for content validity. Psychometric analyses used confirmatory factor models and item response theory (IRT) analyses with particular focus on item fit and item information functions. A novel approach was developed to present qualitative and quantitative evidence to a group of service users, clinicians and researchers to make the final selection. Internal consistency was assessed using Cronbach’s alpha. Known group differences were calculated to test whether the scale was able to discriminate between groups with pre-specified hypotheses. Sensitivity to change was measured between baseline and follow-up for a group of people attending services. The ReQoL was compared with EQ-5D and SWEMWBS.

**Results:** Qualitative evidence from over 90 service users was used to reduce the number of items before field-trial quantitative data were collected from over 6200 service users. Factor analyses supported a unidimensional model. Evaluation of IRT test information functions suggested that a 10 item scale could cover as wide a measurement range as the pool of 40 items. A Cronbach alpha of 0.92 showed good internal reliability. The scale showed moderate responsiveness to change with a standardised response mean of 0.4 for those whose health improved and worsened. The ReQoL was able to detect known group differences and compared well with other measures.

**Conclusion:** This work provides a pragmatic yet rigorous approach to combining qualitative and quantitative evidence to develop the ReQoL to be both psychometrically robust and likely to have high face validity with service users and clinicians.

### Session 1b. Qualitative evidence

#### A59 Development of a novel patient reported outcome measure for patients with haematological malignancy: a qualitative study

##### Pushpendra Goswami^1^, Sam Salek^1^, Tatyana Ionova^2^, Esther Oliva^3^, Adele K Fielding^4^, Marina Karakantza^5^, Saad Al-Ismail^6^, Graham P Collins^7^, Stewart McConnell^5^, Catherine Langton^5^, Daniel M Jennings^8^, Roger Else^9^, Jonathan Kell^10^

###### ^1^School of Life and Medical Sciences, University of Hertfordshire, Hatfield, Hertfordshire, UK; ^2^National Medical Surgical Centre and Multinational Centre for Quality of Life Research, St Petersburg, Russia; ^3^Haematology Unit, Azienda Ospedaliera, Reggio Calabria, Italy; ^4^UCL Cancer Institute, London, UK; ^5^Leeds Teaching Hospitals NHS Trust, Leeds, UK; ^6^Singleton Hospital, ABM University Health Board, Swansea, UK; ^7^Oxford University Hospitals NHS Trust, Oxford, UK; ^8^Royal Surrey County Hospital NHS Foundation Trust, Guildford, Surrey, UK; ^9^Patient Research Partner, Milton Keynes, UK; ^10^Cardiff and Vale University Health Board, Cardiff, UK

####### **Correspondence:** Pushpendra Goswami

**Aims:** The impact of haematological malignancies (HM) and its treatment on patients’ health-related quality of life (HRQoL) is still not well understood. The aim of this study was to identify HRQoL issues and symptoms in patients with HM.

**Methods:** In a UK based multicentre cross-sectional prospective study, adult patients with various HM were recruited from six hospitals in England and Wales. The study employed semi-structured face-to-face interviews with open-ended questions related to the impact of HM and its treatment on HRQoL and symptoms. All the interviews were audio recorded, transcribed verbatim and content analysed using NVivo 11, a qualitative data analysis software.

**Results:** 129 patients (male = 76; mean age = 61.1 years; SD = 15.3; median age =64.92 years; and age range =18-88 years) with mean duration of the HM of 3.6 years (SD = 4.3; and range = 19 days-23 years) were interviewed. Diagnoses were: Acute Myeloid Leukaemia (18); Acute Lymphoid Leukaemia (7); Chronic Myeloid Leukaemia (12); Chronic Lymphocitic Leukaemia (11); Aggressive Non-Hodgkin’s Lymphoma (17); Indolent Non-Hodgkin’s Lymphoma (14); Hodgkin’s Lymphoma (11); Multiple Myeloma (21); Myeloproliferative Neoplasms (10); and Myelodysplastic Syndromes (8). The most prevalent QoL issues important to HM patients were: eating and drinking habits (117); impaired social life and participatory function (86); impaired physical ability or independency (70); disturbed sleep (66); and impaired psychological well-being (64). The most prevalent disease symptoms were tiredness (66); feeling unwell (28); breathlessness (24); lack of energy (21); and back pain (18). Most prevalent treatment side effects were: tiredness (74); feeling sick (36); lack of energy (20); taste disturbance (20); and breathlessness (16)’.

**Conclusion:** The findings clearly indicate that HMs significantly affect patients’ QoL, which is not captured in a systematic manner in routine clinical practice. This highlights the need for the development and validation of a new HM-specific PRO measure for use in such settings.

#### A43 “Normal is redefined”; reflections on outcomes for women with breast cancer: an ethnographic study in London

##### Helen Ward^1^ & Sophie Day^1,2^

###### ^1^Imperial College London, London, UK; ^2^Goldsmiths College, London, UK

####### **Correspondence:** Helen Ward

**Background:** Prognosis for breast cancer is improving with 78% of women surviving >10 years. We conducted an ethnographic study of breast cancer care which focused on experience rather than outcomes, but revealed relevant themes.

**Aim:** To identify outcomes valued by women with breast cancer.

**Methods:** Secondary analysis of ethnographic data including 79 interviews (patients, staff, carers).

**Results:** Women referred to different concerns, preferences and aspirations. Desired outcomes were affected by the experience of treatment, changed over time, and influenced decisions about care. Three major themes emerged.

- Dependency: the dominant theme was a determination not to become dependent, and to continue to care for others. Women described children, partners and parents who they ‘took care of’. This priority affected how they hoped to balance quality and quantity of life; some chose less aggressive treatments in order to have a “decent quality of life to carry on”, others chose radical treatments to maximise their chance of ‘staying around’.
- Normality: some women looked forward to ‘getting back to normal’ but others recognised that they would never be the same again. One described how difficult it would be to resume her previous life, “you are changed, recalibrated, normal is redefined”. Specific aspirations included looking forward to your hair growing back, returning to work, attending a wedding, kayaking, a grandchild starting university. Some women worried about work, asking how they would cope financially, and how they could avoid pity.
- Reciprocity: In addition to some who planned new careers supporting people with cancer, several women aspired to ‘give back’ through volunteering, fund-raising and taking part in research.

**Conclusions:** These themes illustrate the complexity of defining standard patient reported outcomes. We should recognise and respond to evolving preferences and priorities of individuals, avoiding a narrow focus on outcomes defined by other patients.

#### A16 Examining the relevance of PROMs to patients: A review of qualitative data capturing which HRQoL domains are important to patients

##### Elizabeth Lumley^1,2^, Patrick Phillips^2, 1^, Rosie Duncan^1^, Helen Buckley-Woods^1^, Ahmed Aber^1^, Gerogina Jones^3^ & Jonathan Michaels^1^

###### ^1^The University of Sheffield, Sheffield, UK; ^2^Sheffield Teaching Hospitals NHS Foundation Trust, Sheffield, UK; ^3^Leeds Beckett University, Leeds, UK

####### **Correspondence:** Elizabeth Lumley

**Background:** Patient reported outcome measures (PROMs) allow measurement of outcomes elicited from patients; therefore PROMs should include domains that are relevant to patients. One source of this information may be existing qualitative research describing patient experiences and their impact on quality of life (QoL).

**Aims:** The aim of this qualitative evidence synthesis was to examine the symptoms and QoL domains that are important from the perspective of a patient with varicose veins (VV), and compare them to existing PROMs domains.

**Methods:** Eight electronic databases were searched to identify qualitative research published in English of the experiences of adults with VV. A thematic analysis was conducted and resulting themes were compared to existing VV PROM domains to evaluate if they captured the impact the VV have on patients.

**Results:** A total of 1804 citations were identified; after screening only three studies met the inclusion criteria. Five overarching themes were described in the studies; physical impact, psychological impact, social impact, adapting to VV and reasons for seeking treatment.

**Discussion:** The range and intensity of reported symptoms and the participant’s experience of VV was very varied. One key theme to emerge was adaptation, as there was evidence that patients attempted to adapt to the physical, psychological and social impact of VV. No PROM currently exists that would capture how VV patients adapt their lives.

**Conclusion:** The use of PROMS to gather information is well established in the NHS but those currently used may not capture the full impact. Qualitative research methods allow an in-depth understanding of the range and severity of symptoms experienced by patients, and the impact these may have. Dimensions of PROMs should be based on patient experiences, best generated by qualitative research methods.

#### A71 Perspectives of patients and professionals on the use of patient-reported outcome measures in primary care: a systematic review of qualitative studies

##### Ian Porter^1^, Jaheeda Gangannagaripalli^1^, Antoinette Davey^1^, Ignacio Ricci-Cabello^2^, Kirstie Haywood^3^, Stine Thestrup Hansen^4,5^ & Jose Valderas^1^

###### ^1^University of Exeter Medical School, Devon, UK; ^2^Nuffield Department of Primary Care Health Sciences, University of Oxford, Oxfordshire, UK; ^3^Royal College of Nursing Research Institute, University of Warwick, Warwickshire, UK; ^4^Department of Haematology, Zealand University Hospital, Køge, Denmark; ^5^Department of Regional Health Research, University of Southern Denmark, Odense, Denmark

####### **Correspondence:** Ian Porter

**Background:** Although the use of patient-reported outcome measures (PROMs) in healthcare settings has increased substantially over recent years, there is potential for them to play a greater role in primary care.

**Aim:** The underlying aim was to review and summarise studies exploring patients’ and health professionals’ perspectives on the use of PROMs in primary care, to identify positive and negative factors associated with their use, along with barriers and enablers.

**Methods:** A qualitative systematic review was conducted in Medline, Embase, PsychInfo and CINAHL from inception until 2016; further relevant references were retrieved through snowballing. Eligible studies were conducted in primary care settings, using qualitative methods, exploring patients and/or healthcare professional’s perspectives on the clinical utility of using PROMs in clinical practice.

**Results:** 19 studies met the inclusion criteria (4 after 2012), 11 of which were conducted in the UK, reporting on the views of professionals (8), patients (5), and both (7). The majority of studies (12) focused on mood disorders. Patients identified benefits associated with PROMs such as increased awareness, alertness, self-management, feeling validated, and being able to discuss problems they would not otherwise mention. Concerns were raised about PROMs replacing face-to-face consultations, restricting discussion, along with worries about stigma and being identified by illness(es). Professionals reported PROMs to be useful for aiding clinical decisions, monitoring and management options, aiding a better understanding of the longitudinal life of patients. Although PROMs were valued for facilitating communication it was also noted that they undermined the human element of consultations, along with professional intuition and judgement. Burden on GP time was also noted.

**Conclusions:** Patients and professionals highlighted a number of benefits of using PROMs in clinical practice, particularly in terms of supporting decision making, patient awareness and management/self-management options. However, concerns were voiced about PROMs potentially undermining relational continuity of care.

### Session 1c. PROMs for specific patient groups

#### A53 Subjective Quality of Life (QOL) accounts. Essential for applied health research into long term conditions (LTC’s)

##### Deb Roberts

###### Royal Liverpool & Broadgreen University Hospitals Trust, Merseyside, UK

**Background:** This study focuses on QOL measurement in Chronic Fatigue Syndrome (CFS/ME). A heterogeneous and complex illness with fluctuating and unpredictable symptoms.

**Aims:** To pilot a nationally recognised QOL Patient Reported Outcome Measure (PROM) to improve recording and analysis of patients’ personal accounts. To produce accessible data for clinical and research settings, with direct relevance to patient care and commissioning decisions.

**Design and Methods:** A scoping exercise appraised current practices and reviewed relevant measures. By CFS/ME specialist consensus, the World Health Organisation’s QOL PROM of 26 items (WHOQOLBREF-26) was completed with ten patient volunteers. Following a positive professional evaluation, the initiative was registered as a host trust Improvement, Quality and Efficiency project (QEP). A sample of one hundred patients completed the questionnaire in addition to the recommended PROM’s 1. before treatment and 2. after attending group sessions, at the start of individual therapy contact. Quantitative analysis compared longitudinally and to existing tools results.

**Results:** Standard practice, advocated by the British CFS/ME Association of Professionals (BACME) does not fully reflect patients’ self-reporting of CFS/ME. The literature emphasised QOL PROM’s but failed to reveal a universally agreed CFS specific measure. The popular Short Form 36 items (SF36) was regarded as more of a health status tool. Therapists observed ease of administration and interpretation. Change sufficient to endorse the NICE Guidelines for CFS/ME rehabilitation, enhance therapeutic encounters and commissioning decisions was demonstrated. The project outcomes are comparable to other research in this field.

**Conclusion:** Well validated PROM’s are applied in CFS practice and research but exhibit wide variation and lack sensitivity. Although the sample is modest, the WHOQOLBREF-26 provides a quantifiable perspective of the impact of CFS/ME for applied research and clinical application. It shows transferability to the research and evaluation of care in other LTC’s.

#### A33 Effects of Out-of-Pocket (OOP) Payments and Financial Distress on Quality of Life (QoL) of People with Parkinson’s (PwP) and their Carers

##### Anil Gumber

###### Centre for Health and Social Care Research, Sheffield Hallam University, Sheffield, UK

**Background:** Parkinson’s, a neurodegenerative disorder largely affecting the older population, develops into gradual loss of both motor and non-motor functions, thus impacting their daily living activities.

In the UK, over 127,000 people and families are affected by Parkinson’s. Evidence shows an increased financial burden on families who are managing and caring for PwP.

**Aims:** To explore effects of OOP expenditure and loss of income on quality of life and wellbeing of PwP and their caregivers in the UK.

**Methods:** The on-line survey collected information from 853 PwP and caregivers across the UK on changes in their economic, social, health and living conditions due to Parkinson’s during 2015–16. Multivariate regression analyses were attempted, after adjusting for socio-demographic variables, to estimate the net effect of OOP expenditure and income loss on QALYs, VAS and wellbeing scores (on life-satisfaction, life worthwhile, happiness and anxiousness) of PwP and their main carers.

**Results:** QALY, VAS and wellbeing scores of PwP were negatively associated with since how long they were diagnosed with Parkinson’s, i.e. disease progression. Mean OOP cost of treatment and care also increased with the duration of Parkinson’s. Significant negative relationships were found between OOP expenses and income loss on QoL of PwP as well as that of their carers. Magnitudes of coefficients were much higher in PwPs having the condition for >15 years. Similar effects but of smaller magnitudes were found on QoL and wellbeing of carers by duration of disease/ how long caring for. QoL was significantly lower in families having annual income < £20,000.

**Conclusions:** The management and care of Parkinson’s has resulted in considerable financial burden on PwP and their families, which further contributed in deterioration in their quality of life and wellbeing. There is a need to include financial distress indicators in PROMs for those having degenerative conditions including cancers.

#### A34 The Impact of Comorbidities on Outcomes of Hip and Knee Replacement: a meta-analysis

##### Bélène Podmore^1,2^, Andrew Hutchings^1,2^, Jan van der Meulen^1,2^, Ajay Aggarwal^1^ & Sujith Konan^3^

###### ^1^London School of Hygiene & Tropical Medicine, London, UK; ^2^The Royal College of Surgeons of England, London, UK; ^3^University College London Hospitals NHS Foundation Trust, London, UK

####### **Correspondence:** Bélène Podmore

**Background:** Joint replacement surgery is one of the most effective interventions leading to considerable improvements in function and quality of life. Increasing numbers of patients with long-term conditions are undergoing joint replacement surgery. It is important to understand how comorbid conditions impact on outcomes of joint replacement.

**Aim:** This meta-analysis investigated the impact of comorbidities on outcomes of hip and knee replacements.

**Methods:** This study sought all studies that assessed the impact of comorbidities on outcomes of hip and knee replacements. We extracted data for the eleven comorbidities that are recorded in the NHS National PROMs programme. We estimated pooled odds ratios (OR) that express the impact of these comorbidities on ten outcomes including function, pain and health-related quality of life. Differences in continuous outcomes were converted to OR following the Hasselblad and Hedges approach.

**Results:** Fifty-six papers were included. Of these, ten papers looked at function, ten at pain and five at quality of life. Measures that were used for function and pain included the Oxford Knee Score, Knee Society Score, ADL limitation and the WOMAC. For quality of life, the SF-12, SF-36 and Health Utilities Index were used. The evidence suggests comorbidities lead to significantly worse outcomes in the short-term (surgical complications, readmissions, short-term mortality). The evidence for an impact of comorbidities on long-term outcomes is weak however, and the pooled results vary across the different outcomes: quality of life (OR ranging from 0.36 to 1.49), function (OR ranging from 0.68 to 1.69), and pain (OR ranging from 1.01 to 1.41).

**Conclusions:** This meta-analysis shows that comorbidities have an impact in the short-term on outcomes relating to the safety of the surgery but that there is little or no evidence that patients with comorbidities benefit less from hip and knee replacement surgery in the long-term than patients without.

#### A79 Extending the use of PROM scores in the Hip and Knee Replacemnt Patient Pathway in the NHS - Enhancing response rates through patient engagement

##### Andrew Price, William Jackson, Nick Bottomley, Michael Philiips, Toby Knightley-Day, David Beard

###### University of Oxford, Oxford, UK

####### **Correspondence:** William Jackson

**Background:** The National Oxford Hip anf Knee PROMs collection for joint replacement is detached from the patients routine NHS care pathway and has a response rate of 60%.

**Aims:** The aim of this study is to examine whether embedding the use of electroinc PROM score collection in the care pathway, creating value for patients, improves repsonse rates.

**Methods:** The Oxford hip and Knee scores were embedded into the clinical pathway. Using a cloud based eplatform scores were used to supoort decision making prior to surgery, to record the base line level of pre-operative symptoms, to monitor early outcome and to offer longer-term surveillance of outcome. PROMs data was shared with patients at all stages, with the format guided by PPI development input. The response rate at 1-year follow-up was recorded. Comparison was made to standard NHS PROMs collection as a control.

**Results:** Patients stated that electronic PROMs collection was a useful addtion to the pathway. Patients used the following channels to record post-operative ePROM scores: tablet, smart-phone, lap-tops and desk-top PC. The response rate for 1-year follow-up Oxford Score was 90% using all electronic collection methods. During the same time period standard NHS PROM collection repsonse rate was 59% in the control arm.

**Conclusions:** PROM scores can be used to enhance the patients pathway for Hip and Knee Replacement surgery in the NHS. Patients enagagement in PROM use resulted in a significant increase in response rate at longer term follow-up.

### Session 2a. PROMs into practice

#### A06 The Musculoskeletal Health Questionnaire (MSK-HQ): a partnership model of implementation to assess acceptability, feasibility and impact

##### Elizabeth Gibbons & Ray Fitzpatrick

###### Nuffield Department of Population Health, University of Oxford, Oxford, UK

####### **Correspondence:** Elizabeth Gibbons

**Background:** The Musculoskeletal Health Questionnaire (MSK-HQ) is a short self-completed questionnaire intended to be broadly applicable to assess the impact of musculoskeletal problems on patient’s daily life. 1 This provides a case study of how to promote adoption of a PROM in the health service.

**Aim:** The aim was to gain insights into how the MKS-HQ is used by early adopters as a resource to support organisational decision making about services and impact on individual patient care.

**Methods:** A partnership approach to implementation was adopted; a collaboration between a charity (ARUK), researchers and *n* = 10 partners providing a range of MSK services. Interim interviews were conducted over a one year period to share emerging experiences, successes and challenges. Interviews were audio recorded and transcribed and framework analysis conducted using NVivo 10.

**Results:** Logistics and feasibility: Several partners experienced delays in implementation due to reconfiguration of services and installation of new clinical systems. Some experienced challenges with negotiating external processing for electronic capture and data governance. Capacity to process and analyse data was variable; some sites utilised external contractors whilst others relied on existing analytical support.

Interpretability: The minimally important difference (MID) was considered fundamental to interpretation and partners explored different methods of presentation of data. Individual item responses were considered valuable and actionable, specifically items related to information needs and self-management.

Acceptability: The MSK-HQ was acceptable to patients; easy to complete and relevant to their condition. A high level of staff engagement and confidence in the measure was evident with some staff reporting a more structured appointment resulting in more time for the delivery of treatment.

**Conclusion:** Despite some challenges, the MSK-HQ appears to be acceptable, feasible and useful for clinical decision making Partnership between charities, academics and local NHS services are a promising mechanism to implement PROMs.

#### A56 When, how and why does PROMs feedback improve clinician-patient communication within the consultation? A realist synthesis

##### Joanne Greenhalgh^1^, Kate Gooding^2^, Elizabeth Gibbons^3^, Chema Valderas^4^, Judy Wright^1^, Sonia Dalkin^6^, David Meads^1^ & Nick Black^5^

###### ^1^University of Leeds, Leeds, UK; ^2^University of Liverpool, Liverpool, UK; ^3^University of Oxford, Oxford, UK; ^4^University of Exeter, Exeter, UK; ^5^London School of Hygiene and Tropical Medicine, London, UK; ^6^Northumbria University, Newcastle-upon-Tyne, UK

####### **Correspondence:** Joanne Greenhalgh

**Background:** It is suggested that the use of patient reported outcome measure (PROMs) in clinical practice can enhance patients’ consultations with clinicians and improve clinical management.

**Methods:** A realist synthesis to explore the contexts in which and processes through which PROMs enable patients to share concerns with clinicians and change clinicians’ communication practices. We identified the ideas and assumptions (theories) underlying how PROMs use was intended to work. Electronic databases were searched and backwards and forward citation tracking were carried out on key systematic reviews. We selected papers relevant to testing our theories and 36 papers were included.

**Results:** PROMs completion prompts patients to engage in self-reflection about their health. Whether PROMs supported or constrained patients in sharing issues with clinicians depended on the structure of the PROM and existing clinician-patient relationship. Clinicians perceived standardised PROMs constrained their relationship with patients and were difficult to incorporate into the flow of consultations. Clinicians avoided using them or adapted the PROM, which may compromise their validity. Individualised PROMs supported consultations by allowing patients to ‘tell their story’ but were less useful as an outcome measure. In oncology, PROMs did not substantially change clinicians’ communication practices; consultations focused largely on symptoms rather than psychosocial issues.

**Conclusions:** It is the process of PROMs completion which helps patients to reflect on their health and raise issues with clinicians. The structure of the PROMs was a key determinant of the extent to which the use of PROMs supported or constrained the clinician-patient relationship. Future research should consider how PROMs support the relationship building function of the consultation, rather than just the information exchange and decision making functions.

#### A51 Converging and diverging views about PROMs: a qualitative study involving patients and clinicians

##### Carol Fawkes^1^, Robert Froud^2,3^ & Dawn Carnes^1^

###### ^1^Barts and the London School of Medicine and Dentistry, London, UK; ^2^Warwick Medical School Clinical Trials Unit, Coventry, UK; ^3^Norges Helsehøyskole, Campus Kristiania, Oslo, Norway

####### **Correspondence:** Carol Fawkes

**Background:** Although the use of Patient Reported Outcome Measures (PROMs) is being advocated increasingly in clinical practice, there has been a lack of input from patients and clinicians into this process. The success of new initiatives rests on engagement from both of these groups.

**Aims:** To explore the views of patients and clinicians concerning the use of PROMs in musculoskeletal healthcare practice.

**Method:** A combination of focus groups and individual interviews were undertaken with patients and clinicians. A topic guide was used to support semi-structured interviews and focus groups which were audio-recorded. All transcripts were transcribed verbatim and analysed using the Framework approach. Themes and sub-themes were identified, and explanatory models were developed to describe the data.

**Results:** Interviews were held with patients (*N* = 18) and clinicians (*N* = 46) from around the United Kingdom. Clinicians and patients recognised how PROMs could facilitate communication between patients and healthcare professionals, and among healthcare professionals, demonstrate the effects of practice, provide the opportunity to contribute feedback about care, and the importance of having a clear statement on data use and security. Patients stressed their willingness to complete PROMs in an electronic format, and the importance of short questionnaires with a quick completion time. They emphasised their reluctance for PROMs to disrupt the consultation process, and willingness to seek IT help if required. In contrast, clinicians expressed concerns about patient burden and patients’ IT capability, language accessibility, and suitability of PROMs for different settings. While reflection on practice was regarded as important by clinicians, dealing with patients’ feedback was regarded as challenging.

**Conclusions:** The clinicians’ concerns about patient burden and IT capability were not reflected by the patients who were willing to use PROMs. Patients welcomed the opportunity to provide feedback about their care.

#### A78 ACHE - The Arthroplasty Candidacy Help Engine - Using PROMs data to identify thresholds for referral in hip and knee repalcment surgery

##### Andrew Price, Jonathan Cook, Helen Dakin, James Smith, Sujin Kang, David Beard & The ACHE Study Team

###### Univeristy of Oxford, Oxford, UK

####### **Correspondence:** Helen Dakin

**Background:** At present there is no evidence to support the use of the Oxford PROM scores to set pre-operative thresholds for referral for hip and knee replacement surgery. Despite this the practice has been widespread in the NHS.

**Objectives/Research Questions:** Can the Oxford Hip/Knee scores (OKS/OHS) be used to set referral thresholds for hip/knee replacement surgery? Does the choice of threshold affect the cost effectiveness of the procedure?

**Methods:** Thresholds for the Oxford scores were calculated, based on a capacity to benefit model linking pre-operative OKS/OHS to the probability of a good outcome – creating an online ACHE tool. Markov models were constructed to assess how the cost-effectiveness of TKA and THA compared with no arthroplasty varies with pre-operative OKS/OHS over a 10-year time horizon from a UK NHS perspective.

**Results:** The absolute threshold for the OHS was 41 and 42 for the OKS. A model was created that used preoperative Oxford scores to estimate the probability of a good outcome allowed – e.g. an OHS of 35 or an OKS of 30 offers a patient a 70% probability of achieving a good outcome. The economic evaluation demonstrated that TKA and THA cost < £20,000 per quality adjusted life-year (QALY) gained for >99.9% of patients who currently undergo surgery. It is cost-effective to conduct TKA on patients with OKS of 43 (95% credible interval, CrI: 43,44) or less and on patients with THA of 45 or less (95% CrI: 44, 45).

**Conclusion:** Using the ACHE tool the Oxford Hip/Knee Scores can be used to assess an individual patients suitability for hip or knee replacement surgery. On a population level both interventions are highly cost-effective right up to the absolute threshold for intervention. The ACHE tool appears to be a useful evidence based clinical tool to aid referral from primary to secondary care.

### Session 2b. Development and validation of measures – 2

#### A75 Collaborating with burn patients and their families to develop the CARe Burn Scales: a portfolio of burn-specific quality of life PROMs for use in paediatric and adult burn care

##### Catrin Griffiths, Ella Guest & Diana Harcourt

###### University of the West of England, Bristol, UK

####### **Correspondence:** Catrin Griffiths

**Background:** 250,000 people in the UK each year suffer a burn injury. Those affected can experience life-long difficulties including pain, trauma, anxiety, depression and body image distress. Currently there is no standard practice for collecting PROM data in the NHS Burns Service. Few burn-specific PROMs exist and even less have been developed with burn patients themselves. In collaboration with adolescents and adults with a burn injury, family members and health professionals (HPs), we developed the CARe Burn scales: a portfolio of quality of life burn-specific PROMs for use in child, young people, and adult burn care.

**Aim:** To incorporate the opinions of patients and family members in the development of the CARe Burn Scales.

**Method:** Semi-structured interviews were carried out with patients (*n* = 19), family members (*n* = 25) and health professionals (*n* = 8). Thematic analysis identified a number of issues experienced by individuals who are living, or supporting someone, with a burn-injury. The findings informed item generation and the conceptual framework for each PROM. Two systematic reviews of the PROMs currently used in paediatric and adult burn care were also conducted to inform the PROM development. Three age-specific PROMs for children (aged under 8), young people (aged 8–17) and adults (aged 18 and over) were developed. Twenty cognitive-debriefing interviews were then conducted with patients, family members and HPs who reviewed the PROMs in order to identify relevance and readability.

**Results:** The need for burn-specific PROMs to identify patient needs in burn care was reinforced, revisions to improve the PROMs were made and HPs suggested how they would utilise the PROMs within their practice. Psychometric analysis of the PROMs were then conducted using Rasch and classical test theory to further refine and validate the PROMs.

**Conclusion:** The CARe Burn Scales are now available for use in research and clinical practice.

#### A05 Testing the face validity and acceptability of the Primary Care Outcomes Questionnaires through cognitive interviews

##### Mairead Murphy, Sandra Hollinghurst & Chris Salisbury

###### University of Bristol, Bristol, UK

####### **Correspondence:** Mairead Murphy

**Background:** The Primary Care Outcomes Questionnaire (PCOQ) is a new patient-reported outcome measure designed specifically for primary care. It has two formats: the PCOQ-status (which has an adjectival scale) and the PCOQ-change (which has the same items as the PCOQ-status, but a transitional scale).

**Aim:** This paper describes the process of improving the face validity and acceptability of the PCOQ-status and PCOQ-change through cognitive interviews with primary care patients.

**Methods:** Three rounds of cognitive interviews were held with twenty patients from four health centres in Bristol. Tourangeau’s model of cognitive processing was adjusted for this research and used to identify problems. This contained four categories: general comprehension, temporal comprehension, decision process, and response process. The resultant pattern of problems was used to assess whether the items and scales were working as intended, and to make improvements to the questionnaires.

**Results:** The problems identified in the PCOQ-status reduced from 41 in round one to seven in round three. The PCOQ-status was both acceptable to patients and had face valid. As with previous studies, there were very few decision problems, and most comprehension problems. The new category of temporal comprehension helped improve the face validity. The PCOQ-change was less acceptable to patients and was misunderstood, resulting in it being completed incorrectly by 50% of patients. It was not taken forward after round one.

**Conclusions:** Overall the method of cognitive interviewing proved successful in reducing the number of problems for the PCOQ-status identified in each round. The PCOQ-change was poorly understood. Given that this corroborates existing research, this may call into question the use of transitional questionnaires for measurement of outcome in primary care, and the highlights need for cognitive testing of such questionnaires prior to quantitative psychometric testing.

#### A08 Validation and refinement of the HASMID questionnaire: a measure of health and self-management in diabetes

##### Jill Carlton, Jackie Elliott & Donna Rowen

###### University of Sheffield, Sheffield, UK

####### **Correspondence:** Jill Carlton

**Aims:** Last year we presented the processes undertaken to develop a new questionnaire to evaluate self-management in diabetes (HASMID questionnaire). This new study aims to assess the 1) mode of administration (online and paper-based) and 2) psychometric properties of HASMID to explore the relationships between some items and further refine the instrument.

**Methods:** Focus groups were held with people living with diabetes to further reflect on wording of the original 8-item HASMID questionnaire. Alternative phrasing was suggested for 5 items covering mood, daily routine, stress and hassle, and all 13 items were put forward for validation. The validation study consisted of two arms/groups - online or paper survey. Respondents were asked to complete 13 HASMID questions, EQ-5D and sociodemographic questions. Potential participants were recruited via a patient database and social media (email and Twitter), and asked to complete the survey at two time points, so that the responsiveness of the HASMID instrument could be assessed.

**Results:** Data collection for time point 1 is ongoing. Recruitment (up to 1st February 2017) included 1756 and 865 completions to the online and paper versions respectively. Completed responses from people with diabetes included those with type 1 = 792, type 2 = 1980 and other forms of diabetes = 49. Data collection for time point 2 will be complete in May 2017. Results will be presented to demonstrate the reliability (internal consistency) and validity (known-group differences) of the HASMID instrument. Item correlations, missing data, floor and ceiling effects, and comparison with EQ-5D scores will be reported. Reference will be made to the quality of data collected via paper vs. online completes. The refined HASMID questionnaire will be presented.

**Conclusions:** This study will demonstrate the benefits of further validation in the development of instruments. In addition, the consideration of differing modes of administration, and participant recruitment will be discussed.

### Session 2c. Analytic issues

#### A61 What exactly is a good outcome for total knee replacement (TKR)? And what proportion of patients experience one?

##### Anqi Gao, Andrew Price & David Beard

###### University of Oxford, Oxford, UK

####### **Correspondence:** Anqi Gao

**Background:** Patient reported outcome measures (PROMS) are used to assess total knee replacement (TKR). Variation exists in reported values for the proportion of patients achieving a “good outcome”, ranging from 53%-79%. The variation could reflect how a “good outcome” is calculated rather than true differences in study populations.

**Aim:** Using a large data set, the aim of this study is to determine the proportion of THR and TKR patients experiencing a “good outcome” using different criteria, and compare the results obtained via each method.

**Methods:** The TKR-PROMs dataset (2012–2013, *n* = 40,622), including 6-month Oxford Knee Scores (OKS) was investigated. Four common criteria (shown below) were used to calculate the proportion of patients achieving a good outcome:

1. ½ standard deviation (SD): For good outcome, the change pre- to post-operative OKS (ΔOKS) must reach ½ SD of the average baseline OKS for the population
2. Minimally Important Change (MIC): For good outcome, ΔOKS must reach the MIC.
3. Satisfaction Criteria: Good outcome defined by ΔOKS or 6-month post-op OKS must reach the patient acceptable symptom state (PASS) score, which separates the satisfied from the unsatisfied.
4. Response: Good outcome defined by modified OMERACT-OARSI criteria, which categorizes patients as “responders” and “non-responders” based on change in pain and function.

**Results:** Good outcome was achieved in 88% (½ SD), 74% (MIC), 71% (Satisfaction/PASS) and 83% (Response/OARSI) respectively.

**Conclusions:** In the same population, the proportion of patients achieving a good outcome can appear different depending on the metric used. Caution should be applied when viewing the outcome of data sets using different methods, particularly when using binary thresholding methods. A universal method of calculating and describing good outcome should be developed.

#### A04 Symptom clusters for revising scale membership in the analysis of Patient Reported Outcome Measures

##### Agnieszka Lemanska^1^, Tao Chen^1^, David P Dearnaley^2^, Rajesh Jena^3^, Matthew Sydes ^4^ & Sara Faithfull^1^

###### ^1^Univerity of Surrey, Guildford, UK; ^2^Institute of Cancer Research and Royal Marsden NHS Trust, London, UK; ^3^Cambridge University Hospitals, Addenbrookes Hospital, Cambridge, UK; ^4^MRC Clinical Trials Unit at UCL, Institute of Clinical Trials and Methodology, London, UK

####### **Correspondence:** Agnieszka Lemanska

**Background:** Summative scores (individual items pooled together usually using average) are used to extract and interpret information contained within PROMs. Tools such as University of California, Los Angeles Prostate-Cancer-Index (UCLA-PCI) arrange items in scales according to the underlying health concern. Those scales are used to create agglomerate scores. However, this generic approach may be less sensitive e.g. for varying side-effect profiles due to different treatments. An alternative method that offers a flexible and adaptive approach of grouping symptoms and summarising PROMs is to use symptom clusters (SC).

**Aims:** To compare SC with scales. To explore the value of SC in the analysis and interpretation of PROMs and in symptom management for patients.

**Methods:** A dataset of 843 prostate cancer patients from a randomised control trial called RT01 was used. PROMs were reported with the UCLA-PCI. SC were explored with hierarchical cluster analysis (HCA) (correlation > 0.6). Spearman’s correlation was used to investigate the strength of the relationship between the items in the UCLA-PCI and Cronbach’s Alpha to evaluate the reliability of the Urinary Function Scale.

**Results:** Two SC were identified (Urinary Cluster and Sexual Cluster). The grouping with SC was different than scales. Two items of the Urinary Function Scales (“Number of pads” and “Urinary leak interfering with sexual activity”) were not included in the Urinary Cluster due to the low correlation which ranged from 0.20-0.21 and 0.31-0.39 respectively. Cronbach’s Alpha also showed low correlation of those items with the scale (0.14-0.36 and 0.33-0.44 respectively). All Urinary Function Scale items were subject to a ceiling effect.

**Conclusion:** SC provide a study specific approach for adaptive modelling and interpretation of PROMs. Multiple-item scales should be evaluated with SC.

#### A22 Mapping between PROMS, and the Relative Responsiveness of PROMS : a meta-analytic approach

##### AE Ades, Daphne Kounali & Guobing Lu

###### University of Bristol, Avon, UK

####### **Correspondence:** AE Ades

**Background:**

There is often a need to synthesise treatment effects across trials which have reported similar but different outcomes. Standardised Mean Differences are used for this, but this introduces random error. There is also interest in the relative responsiveness of test instruments, and in the relationships between treatment effects on different - but similar - scales, sometimes for the purpose of health economic evaluation. The purpose of this research was to develop methods that achieve all these aims.

**Methods:**

Statistical methods were developed to synthesise information on different measurement scales both within and between trials, from connected networks of trials. (“Connected” here means that each trial includes at least one test instrument that was used in at least one other trial). A key assumption was that the larger the treatment effect on one scale, the larger on the other scales. The models estimated coherent “mappings” between test instruments. If applied to standardised effects, the mappings reflect relative responsiveness. These models were applied to 3 datasets: first to 6 outcomes from 8 trials on biologic therapies in Ankylosing Spondylitis; second to 9 outcomes from 22 trials on treatments for Social Anxiety; and third to 6 outcomes on 19 trials of treatments of depression.

**Results:**

The models fitted the data well, although the mappings varied slightly from trial to trial with a coefficient of variation approximately 13%. This may reflect non-linearity in the relationships between scales. Models based on standardised mean differences fitted less well, and estimated effects with less precision. It was possible to rank scales in responsiveness, and identify some scales as clearly more responsive than others.

**Conclusions:**

Simultaneous synthesis and estimation of mappings is superior to standardisation while making fewer assumptions. It provides insights into the relative responsiveness of test instruments, and can efficiently estimate “mappings” between outcome scales.

#### A09 To impute or not to impute? A comparison of statistical approaches for analysing missing longitudinal patient reported outcome data in randomised controlled trials

##### Ines Rombach, Alastair Gray, Crispin Jenkinson & Oliver Rivero-Arias

###### Nuffield Department of Population Health, University of Oxford, Oxford, UK

####### **Correspondence:** Ines Rombach

**Background:** Missing data are a potential source of bias in the results of randomised controlled trials (RCTs), but are generally unavoidable in clinical research, particularly in patient reported outcome measures (PROMs). For longitudinally collected outcomes, often only a small subset of participants will have complete data for all relevant time points. Approaches to handling missing longitudinal data include maximum likelihood (ML), multiple imputation (MI) and inverse probability weighting (IPW).

**Aims:** To compare ML, MI and IPW approaches for handling missing longitudinal PROMs data in RCTs.

**Methods:** Realistic missing at random data were simulated using follow-up results from an RCT using the Oxford Knee Score. Simulation scenarios covered sample sizes ranging from 100 to 1,000 with missing PROMs outcome data in 10% to 60% of participants. Different monotone and non-monotone missing data patterns were considered. Missing data were addressed using ML, MI and IPW; data were analysed via multilevel mixed-effects linear regression models. Root mean square errors in the treatment effects were used as performance parameters for 1000 simulations.

**Results:** Non-convergence issues were observed for the IPW approach for small sample sizes. Bias in the treatment effects increased both with decreasing sample size and increasing proportions of missing data. MI and ML performed similarly when the imputation model was restricted to baseline variables. However, MI was less biased when post-randomisation data were used in the imputation model. Both approaches were less biased for non-monotone vs. monotone missing data patterns. IPW introduced more bias than ML and MI across all sample sizes and missing data scenarios.

**Conclusions:** MI can offer benefits over ML for handling missing longitudinal PROMs data when additional post-randomisation information is available. IPW is not recommended to handle missing PROMs data in RCTs with sample sizes of 1000 participants or less. Minimising missing data remains important in reducing bias.

### Session 3a. Electronic delivery and CAT

#### A38 Patients, staff and relatives agree: electronic PROM reporting of adverse events is a feasible and acceptable adjunct to standard care in pelvic radiotherapy for cancer: eRAPID (Electronic patient self-Reporting of Adverse-events: Patient Information and aDvice)

##### Patricia Holch^1,2^, Marie Holmes^2^, Zoe Rodgers^2^, Sarah Dickinson^2^, Beverly Clayton^2^, Susan Davidson^3^, Jacqui Routledge^3^, Julia Glennon^3^, Ann M Henry^4,2^, Kevin Franks^4^ & Galina Velikova^2,4^

###### ^1^Leeds Beckett Universty, Psychology Group, Leeds, West Yorkshire, UK; ^2^Univeristy of Leeds, Patient reported Outcomes Group, Leeds, West Yorkshire, UK; ^3^The Christie NHS Foundation Trust, Manchester, Lancashire, UK; ^4^Leeds Teaching Hospitals NHS Trust, Leeds, West Yorkshire, UK

####### **Correspondence:** Patricia Holch

**Background:** PROMs are routinely used to report late effects of pelvic radiotherapy however recent reviews (Holch 2016 & Gilbert 2015) have shown they are under-utilised in the acute phase. We aimed to explore the acceptability and feasibility of an innovative online self-report and symptom management system (eRAPID) incorporating PROMs into the electronic patient records in routine cancer care. Currently eRAPID is proving acceptable and useful to patients undergoing systemic cancer treatment.

**Aims:** To explore whether radiotherapy patients and their relatives could see the benefits of online PROM reporting despite daily hospital attendance and if staff would find the electronically accessible PROM data a useful adjunct to clinical assessments.

**Methods:** *N* = 73 participants took part in audio-recorded semi-structured interviews at St James’s Institute of Oncology, Leeds and The Christie Hospital, Manchester. Patients (*n* = 37) undergoing radiotherapy/concurrent chemotherapy for prostate, colorectal and gynaecological cancer were interviewed during, on completion and 6 weeks post-treatment (age range 42–81 mean 62).

Additionally we interviewed key staff (nurses, oncologists and radiographers) (*n* = 26) and patient relatives (*n* = 9). Interviews were transcribed, coded thematically and managed in NVivo.

**Results:** Emergent themes revealed patients were positive about the online PROM system. They thought eRAPID could ‘enable the reporting of difficult or challenging symptoms’ and be a ‘source of out-of-hours information’. Staff felt the system would ‘enable support during follow up’ and ‘fill gaps in toxicity reporting’. Relatives highlighted that eRAPID would ‘forewarn of potential side effects’ and has the potential to ‘maintain patient’s independence’.

**Conclusions:** Findings suggest that despite daily hospital attendance both patients and their relatives stated an online PROM management system would be beneficial in the acute and late phases of treatment. Staff agree that eRAPID would be a valuable enhancement to current service provision. A feasibility study is currently underway at Leeds and The Christie NHS Trusts.

#### A24 The eSMART RCT: Comparing electronic Symtpom Management using the Advanced Symptom Management System (ASyMS) with standard care for patients during adjuvant chemotherapy

##### Roma Maguire^1^, Lisa McCann^1^, Teresa Young^2^, Jo Armes^3^, Jenny Harris^3^, Christine Miaskowski^4^, Grigorios Kotronoulas^1^, Morven Miller^1^, Emma Ream^1^, Elizabeth Patiraki^5^, Alexander Geiger^6^, Geir V. Berg^7^, Adrian Flowerday^8^, Peter Donnan^9^, Paul McCrone^3^, Kathi Apostolidis^10^, Patricia Fox^11^, Eileen Furlong^11^ & Nora Kearney^11^

###### ^1^University of Strathclyde, Glasgow, UK; ^2^East & North Herts NHS Trust, Mount Vernon Cancer Centre, Northwood, UK; ^3^Kings College, London, UK; ^4^University of California, San Francisco, CA, USA; ^5^National & Kapodistrian University of Athens, Athens, Greece; ^6^Medical University Vienna Comprehensive Cancer Centre, Vienna, Austria; ^7^NTNU, Innlandet Hospital Trust, Lillehammer, Norway; ^8^Docobo, Bookham, UK; ^9^University of Dundee, Dundee, UK; ^10^European Cancer Patient Coalition (ECPC), Brussels, Belgium; ^11^University College Dublin School of Nursing, Dublin, Ireland

####### **Correspondence:** Teresa Young

**Backgound:** ASyMS is a mobile-phone based remote-monitoring and alert system enabling real-time monitoring of patients’ chemotherapy-related symptoms. The eSMART RCT compares patient reported outcomes (PROs) and delivery of care for patients using ASyMS with those accessing the same Acute Oncology Service through the standard care pathway at each participating site.

**Methods:** Patients newly diagnosed with breast, haematological or colorectal cancer and scheduled to receive at least three cycles of chemotherapy are recruited into this multinational repeated measures, parallel-group stratified RCT. 1108 patients will be randomised to either ASyMS or standard care. Patient demographic and clinical data is collected at baseline and standardised PRO measures are completed at baseline, during each cycle of chemotherapy and every 3 months up to 12 months after chemotherapy. The primary outcome measure is the Memorial Symptom Assessment Scale. Secondary measures include: Functional Assessment of Cancer Therapy-General, Supportive Care Needs Survey-Short Form 34, State-Trait Anxiety Inventory, Communication and Attitudinal Self-Efficacy scale for cancer, Work limitations Questionnaire, EuroQol and Client Services-Receipt Inventory.

**Results:** RCT data collection started in May 2016, and to date 432 patients have been recruited from 11 centres in 5 countries including participants from the UK.

**Conclusion:** Patients are willing to accept randomisation into the study and recruitment is ongoing. An overview of the trial aims and design and a reflection on the challenges faced in setting up the study and the solutions implemented to overcome them will be provided.

**Acknowledgement**

The study is funded by a grant from the EC: FP7-HEALTH-2013-INNOVATION-1 Grant Agreement Number 602289.

#### A27 Quality of life assessment is more efficient precise when conducted using a computer adaptive testing protocol : an international study

##### Chris Gibbons

###### University of Cambridge, Cambridge, UK

**Background:** We calibrated four item banks using the WHOLQOL-100 quality of life assessment and deployed them online as computer adaptive tests using the Concerto assessment platform.

**Methods:** The WHOQOL-CAT was developed using Concerto hosted online. Item selection and scoring was conducted using the partial credit ‘Rasch’ model with maximum likelihood estimation. The WHOQOL-CAT provides four separate unidimensional assessments of quality of life in physical, psychological, social and environmental domains.

**Results:** 4886 patients from 115 countries, including 578 from the UK, completed the WHOQOL-CAT. 1745 participants reported a long-term condition and 586 participants reported multiple long-term conditions (multimorbidity). Average age was 35 years (range 18 to 92) and 47% of the sample were female. Assessments were conducted with a mean reliability (Alpha Cronbach’s equivalent) of .83 (range .82-.88) using, on average, 4.8 items per domain. Average assessment time was 95 seconds (4.8 seconds per item). Participants who self reported long-term conditions reported significantly lower scores on the physical quality of life domain (t = −3.06, *P* < 0.01) but not on any of the other four domains.

**Conclusions:** When administered to a diverse international sample of participants, the WHOQOL-CAT compares favourably to the WHOQOL-BREF and WHOQOL-100. The WHOQOL-CAT is between 80% and 25% shorter than the paper-based measures and is slightly more reliable. Accurate QoL assessment across four domains can be made in under two minutes. Advantages of CAT which are typically demonstrated using simulated CAT administration ‘in the lab’ appear to be evident in studies using data collected ‘in the real world’ from a diverse international sample.

#### A23 The PROMIS Profile 29 allows valid comparisons of patient-reported health over France, Germany and the UK

##### Felix Fischer^1^, Chris Gibbons^2^, Joel Coste^3^, Jose Valderas Martinez^4^, Matthias Rose^1^ & Alain Leplege^5^

###### ^1^Charité Universitätsmedizin Berlin, Berlin, Germany; ^2^The Psychometrics Centre, University of Cambridge, Cambridge, UK; ^3^APEMAC, EA 4360, Paris Descartes University and Epidemiology Unit, Hôtel Dieu, Assistance Publique, Hôpitaux de Paris, Paris, France; ^4^University of Exeter Medical School, Exeter, UK; ^5^Département d’Histoire et de Philosophie des Sciences Université Paris Diderot, Paris, France

####### **Correspondence:** Chris Gibbons

**Background:** Patient-Reported outcome measures are increasingly utilized. Comparability of such measures over different languages needs to be established to allow cross-national research. We investigate the comparability of the PROMIS Profile 29, a generic health-related quality of life measure, in general population samples in the UK, France and Germany.

**Methods:** A web based survey was simultaneously conducted in the UK (n = 1,509), France (1,501) and Germany (1,502). Along with the PROMIS profile 29, the participants answered sociodemographic questions as well as the EQ-5D. We tested measurement invariance by means of multi-group confirmatory factor analysis. Differences in the health-related quality of life between countries were modeled by linear regression. We present general population reference data for the included PROMIS domains utilizing plausible value imputation and quantile regression.

**Results:** Multi-group confirmatory factor analysis of the PROMIS Profile 29 revealed strong measurement invariance between different languages (CFI = 0.952, TLI = 0.950, RMSEA = 0.049, SRMR = 0.041) . We observed significant differences in patient-reported health between countries, which could be partially explained by differences in overall ratings of health. The physical function and pain interference scales showed considerable floor effects in the normal population in all countries.

**Conclusion:** Scores derived from the PROMIS Profile 29 can be readily compared across the UK, France and Germany. Due to the use of plausible value imputation, the presented general population reference values can be compared to data collected with other PROMIS short forms or computer-adaptive tests.

### Session 3b. Concepts and models

#### A12 The Development and Use of Conceptual Models

##### Sarah Shingler, Natalie Aldhouse, Tamara Al-Zubeidi, Andrew Trigg & Helen Kitchen

###### DRG Abacus, Oxfordshire, UK

####### **Correspondence:** Sarah Shingler

**Background:** Conceptual models (CMs) provide visual representation of disease related concepts and their causal linkages. CMs can aid the development and/or revision of patient reported outcome (PRO) instruments; guiding decisions around what and how to measure (e.g. identification of clinical trial endpoints and suitable PROs).

**Aims:** To undertake a targeted review of the use and development of CMs in published PRO research, using examples to make suggestions for best practice for developing and using effective CMs.

**Methods:** A targeted literature search was undertaken in Pubmed (2007–2017) for publications associated with CM development associated with PROs. A separate hand search was conducted in relevant conference websites, such as ISPOR to identify CM guidelines and/or publications. Data was reviewed for CM development methods to guide best practice suggestions.

**Results:** Of 47 abstracts identified and reviewed, 12 were found to be potentially relevant for full text review; one didn’t include a CM and was therefore excluded. Five of the included publications included patient research (qualitative interviews/focus groups) in their CM development; another five only included information extracted from a literature review (qualitative patient data), and one lacked development details. The majority of CMs included domains and sub-domains associated with symptoms, impact on health related quality of life, and associated treatment impacts. Only three studies reported including data from/review by clinical experts. No best practice guidelines for CM development were identified; although publications specific to PRO content validity were. These state that qualitative data should be included in research to confirm the conceptual framework of PROs used in trials.

**Conclusions:** CMs are important for identifying disease concepts from the patient perspective which is essential for identifying trial endpoints and confirming PRO content validity; a necessity for regulators. For a CM to fully explore patient perspective, the inclusion of patient data during the development appears to be essential.

#### A55 Chronobiology and PROMs: a systematic scoping review for patients with chronic health conditions

##### Antoinette Davey, Ian Porter, Colin Green & Jose M Valderas

###### University of Exeter, Exeter, UK

####### **Correspondence:** Antoinette Davey

**Background:** Aggregate estimates of Patient Reported Outcome Measures (PROMs) are being increasingly used for measuring health care providers’ performance. There is also growing recognition of the potential of the individual use in enhancing patient centredness in the provision of clinical care. The impact of biological, environmental and social cyclical phenomena on intra-individual PROMs estimates has previously received little attention because of the implicit assumption that it may be negligible for group-level estimates. It may be however significant for individual use.

**Aims:** To map the key concepts that underpin research in the chronobiology of PROMs as well to identify and summarize available evidence for patients with chronic conditions.

**Methods:** We conducted a systematic search and review of the literature, following the PRISMA statement and a protocol in the PROSPERO database. Electronic databases (MEDLINE, Embase, PsychInfo, and CINHL) were searched using MeSH terms, free text and previously developed search strategies. All documents literature for chronobiology and PROMs focusing on chronic conditions were included, without any restriction. Two reviewers independently applied the criteria in the consecutive stages of title, abstract and full-text screening. A narrative synthesis of the data will be conducted.

**Results:** 2098 unique titles were identified, the majority of them (1460, 69.6%) were published in the last 10 years. The review is currently in progress and full results will be ready to report at the conference. Literature consists of quantitative analyses of observational data, and reports from experimental studies. Evidence have been identified for daily variations of patient reported outcomes as a result of biological rhythms for a range of chronic conditions, such as arthritis, asthma, migraines, depressive symptoms and cognitive status.

**Conclusions:** If chronobiology and its effects can be identified and therefore considered in a policy setting this will facilitate further use of PROMs in an individualised patient setting.

#### A76 Diversity in values across the life-course

##### Joanna Coast^1,2^

###### ^1^University of Bristol, Bristol, UK; ^2^NIHR CLAHRC West, Bristol, UK

**Background:** This paper considers the possibilities for the application of capability measures within a life course framework. Life course concepts focus on interlinking between generation and age. This may be a particularly useful way to think about values within a capability framework, given the importance of context within the approach and the potential for diversity in considering capability to different groups.

**Aims:** To explore the extent to which ‘what people have reason to value’ differs at different points in the life course.

**Methods:** (i) Examination of existing capability measures for adults, older people and end of life, generated using similar methods (in-depth interviews with the populations of interest and valuation using best-worst scaling/discrete choice techniques) to determine similarities and differences in values at different points of the life-course; (ii) Examination of issues that would arise in fully exploiting a life-course approach to capability measurement.

**Results:** Measures for adults and older people are broadly similar but there are important differences that may reflect life course issues, particularly in relation to differences between Achievement (adults) and Role (older people), and between Stability (adults) and Security (older people). The end of life measure has much greater variation within its attributes, appearing to reflect concerns with anticipated lack of capacity at end of life. Important issues in generating a comprehensive life-course approach include delineating points on the life course at which values changes, integrating measures across different stages of life, and generating appropriate measures for children that capture both well-being and well-becoming, and rapid changes in development and capacity.

**Conclusions:** Despite many challenges, a life-course approach to capability measurement may well offer an approach that accounts for much of the diversity in values without requiring new capability indices for every situation; pursuing this approach would be a worthwhile endeavour.

#### A44 Understanding DEMQOL scores: Minimal Important Differences

##### Sarah Smith, Jolijn Hendriks & Nick Black

###### London School of Hygiene & Tropical Medicine, London, UK

####### **Correspondence:** Sarah Smith

**Background & Aims:** DEMQOL (a measure of HRQL in dementia) has undergone extensive psychometric evaluation but no minimal important difference (MID) has been reported. MID statistics are enhanced by qualitative, descriptive statements about what it means to change from one score to another over time.

**Methods:** In a sample of 875 patients attending a first Memory Assessment Clinic appointment and 6 month follow up, we used anchor-based (mean score difference between those reporting “no difference” and those reporting “a little” difference in HRQL) and distribution-based (effect sizes of 0.5) to quantify MID. We developed three example scenarios of MID change and identified the item descriptions related to this change.

**Results:** Anchor-based and distribution-based methods produced different estimates of MID (1.92 v 6.15 respectively). Given the known difficulties in interpreting the former we used the distribution-based statistic to develop descriptive item scenarios.

People with dementia at the high end of the HRQL scale (score >66) are most likely to report low negative emotion (‘a little’ worried, frustrated, and fed up). In the middle of the scale (56) participants also report worry about cognitive function (‘a little’ worried about forgetting who people are and having difficulty making decisions; ‘quite a bit’ worried about forgetting things that happened recently). At the low end of the scale (38) participants additionally report greater worry about social situations (‘a lot’ worried about how they get on with others and making themselves understood). In general, a 6 point (MID) change for each of these scenarios resulted in descriptive changes equivalent to one response category.

**Conclusions:** Using Rasch analyses to develop the scores for DEMQOL has given us important information about the location of each item on the continuum. We have linked this information to MID statistics to begin to develop a meaningful understanding of change.

This research was commissioned and funded by the Department of Health Policy Research Programme (Using Patient Reported Outcome Measures to Assess Quality of Life in Dementia, 0700071). The views expressed in this publication are those of the authors and not necessarily those of the Department of Health.

### Session 3c. Preference - based measures

#### A10 Use of PROs in children: development of a value set for the EQ-5D-Y

##### Koonal Shah^1,2^, Oliver Rivero-Arias^3^, Juan-Manuel Ramos-Goni^4^, Simone Kreimeier^5^, Mike Herdman^1^ & Nancy Devlin^1^

###### ^1^Office of Health Economics, London, UK; ^2^University of Sheffield, Sheffield, UK; ^3^University of Oxford, Oxford, UK; ^4^EuroQol Office, Rotterdam, The Netherlands; ^5^University of Bielefeld, Bielefeld, Germany

####### **Correspondence:** Koonal Shah

**Background:** There are challenges in measuring self-reported health in children, and in valuing the resulting health states. For example, whose preferences should be used to value the health states defined by a preference-based measure designed to measure children’s health and to provide utilities for economic evaluation? The EuroQol Group has developed a child-friendly version of the EQ-5D - the EQ-5D-Y - but to date there have been no value sets available to support its use in decision-making.

**Aims:** (i) To provide an overview of the challenges in defining and valuing children’s health states; (ii) to report a discrete choice experiment (DCE) study undertaken to obtain latent scale values for the EQ-5D-Y; and (iii) to report a methodological study, undertaken in parallel, to explore and compare a range of stated preference methods for valuing EQ-5D-Y health states. The ultimate aim is to provide an EQ-5D-Y value set for the UK, and a set of methods that can be recommended for use in other countries requiring an EQ-5D-Y value set.

**Method:** The DCE was administered via an internet survey. 1,000 adult general public respondents were asked to consider EQ-5D-Y health states from the perspective of a 10-year-old child. The methodological study was administered via computer-assisted personal interviews, and tested four techniques: visual analogue scale, DCE, time trade-off and an innovative ‘location-of-dead’ approach. 300 adult general public respondents were asked to value both EQ-5D health states considering their own health AND EQ-5D-Y health states for a 10-year-old child.

**Results:** This presentation explored the normative and practical challenges involved in valuing child health states and reported the preliminary findings of the empirical research (full results of the DCE study and findings from a pilot of the methodological study).

**Conclusions:** This research has advanced the understanding of the valuation of children’s health outcomes and represents the first stage in the development of EQ-5D-Y value sets.

#### A17 Selection of bolt-ons after factor analysis: are linear regression models a useful technique?

##### Aureliano Paolo Finch, John E Brazier, Clara Mukuria

###### University of Sheffield, Sheffield, UK

####### **Correspondence:** Aureliano Paolo Finch

**Background:** It is recognized that the EQ-5D may miss dimensions important for the HRQoL of patients. When this happens, a possible solution is adding bolt-ons. Finch et al., (2017) have recently shown that bolt-on can be systematically idenfied using factor analysis. This technique pinpoints to a list of factors and items. However, factors and items cannot be added to the EQ-5D simultanously, as this would affect the measure’s feasibility. Hence, it remains unclear how to select bolt-ons from the identified list. This study investigates the possibility of using linear regression models for the selection of bolt-ons after factor analytic identification. It tests six factors, energy/vitality, satisfaction, relationships, hearing, vision and speech, and 37 items loading on them.

**Methods:** Two tests were performed. In the first test, linear regressions were fitted to determine whether different factors and items helped explain variations of self reported health as measured by the VAS health scale. Bolt-on relevance was judged comparing the strength, direction and statistical significance of unadjusted b coefficient. In the second test, linear regressions were fitted to further investigate whether different factors and items helped explain the negative effect of six chronic conditions on self reported health. A reduction in the coefficients for the chronic conditions dummies meant that the factor or item detected the effect.

**Results:** Energy/vitality, relationships and satisfaction reported substantially larger unadjusted b coefficients than speech, vision and hearing. Also items loading on energy/vitality, relationships and satisfaction generally presented larger coefficient than those of items loading on speaking, vision and hearing. The second test did not detect b coefficient decrements for the chronic conditions dummies when testing factors, but consistent b coefficient decrements when testing items.

**Conclusions:** The first test appears a useful technique for bolt-on selection after factor analytic identification. Further research is needed for the second test.

#### A45 New methods for analysing the distribution of EQ-5D observations

##### Bernarda Zamora^1^, David Parkin^1^, Yan Feng^1^, Andrew Bateman^2,3^, Mike Herdman^1^ & Nancy Devlin^1^

###### ^1^Office of Health Economics, London, UK; ^2^Cambridgeshire Community Services NHS Trust, Cambridge, UK; ^3^University of Cambridge, Cambridge, UK

####### **Correspondence:** Bernarda Zamora

**Background:** This paper proposes and tests new methods (Health State Density Index, Health State Density Curve and estimated Power Law functions) we have developed to characterise and summarise the distribution of health states in Patient Reported Outcome (PRO) data within a sample or population of patients. The purpose of these methods is to measure how diverse any given patient group is. These results are relevant to clinicians responsible for service delivery - for example, a homogenous population of patients can be managed by protocol based interventions whilst heterogeneity requires more complex service delivery. The methods also have applications in comparing the descriptive performance of alternative instruments (e.g. EQ-5D-5 L vs EQ-5D-3 L); and in understanding the underlying nature of changes in health states resulting from treatment in clinical settings or trials.

**Methods:** Using both the new methods and existing methods from information theory (e.g. Shannon’s Index), we compare the distribution of EQ-5D health profiles across three groups of patients in two data sets: Cambridgeshire Community Services NHS’s electronic patient records for the EQ-5D-5 L; and the Health Survey for England 2014 for the EQ-5D-3 L. To explore the link between changes in the distribution of self-reported health and health changes, we use data from the NHS PROMs programme which reports EQ-5D-3 L before and after four surgical procedures. The properties of the various methods are further examined using simulated data sets.

**Conclusions:** Our results show that each method has different properties and will give different insights into patients’ data. For example, the Shannon index (absolute and relative) is not sensitive to random variations but decreases slowly with “rare health states”. The HSDI decreases slowly with random variations and is strongly affected by “rare” health states with large decreases towards zero (total inequality). We discuss the implications for the interpretation and analysis of PRO data.

#### A67 Putting the P back into PROMs - using patient valuations of EQ-5D health states to improve hospital performance comparisons

##### Thomas Patton^1^, Nils Gutacker^1^ & Koonal Shah^2,3^

###### ^1^University of York, York, UK; ^2^Office of Health Economics, London, UK & ^3^University of Sheffield, Sheffield, UK

####### **Correspondence:** Thomas Patton

**Background:** The English NHS has been collecting EQ-5D data for all publicly-funded patients undergoing four types of elective surgery since April 2009. One of the intended aims of these data is to inform patients’ choice of hospital. To this end NHS Digital publishes performance indicators reflecting the change in EQ-5D utility scores. These are calculated using the UK TTO general population value set.

While the approach of using general population value sets is justified in many applications, for example when evaluating new technologies for adoption into collectively funded healthcare systems, it appears indefensible when HRQoL data are used to inform individual patients’ decisions about where to receive care.

**Aims:** This study explores whether using patient weights instead of general population weights leads to a) different rankings of hospital providers and b) changes in providers deemed performance outliers.

**Methods:** We use data EQ-5D health state data and EQ-VAS data from over 300,000 patients who underwent hip and knee replacement surgery in England between April 2009 and March 2015 to derive procedure-specific patient population tariffs. We apply these tariffs to the EQ-5D health state data before and after surgery to calculate case-mix adjusted hospital performance estimates. Changes in ranking are calculated for each patient for their 10 closest hospitals. Changes in outlier status are calculated for all providers in England.

**Results:** Patient population tariffs assign lower weight to pain & discomfort and higher weights to anxiety & depression than the UK general population TTO tariff. Hospital rankings vary depending on the weights used. There is little impact on outlier identification.

**Conclusion:** Patients may be better served with hospital performance estimates that reflect their own values and are therefore more relevant to their decisions. This finding has important implications for the national PROM programme in England.

